# Direct Advancement of Balloon-Guide Catheter Distal to the Carotid Free-Floating Thrombus Achieves Smart Mechanical Thrombectomy

**DOI:** 10.7759/cureus.22439

**Published:** 2022-02-21

**Authors:** Hiroyasu Inoue, Masahiro Oomura, Yusuke Nishikawa, Mitsuhito Mase, Noriyuki Matsukawa

**Affiliations:** 1 Neurology, Nagoya City University Graduate School of Medical Sciences, Nagoya, JPN; 2 Neurosurgery, Nagoya City University Graduate School of Medical Sciences, Nagoya, JPN

**Keywords:** ca19-9, acute ischemic stroke, mechanical thrombectomy, tandem lesion, carotid free-floating thrombus

## Abstract

A 68-year-old man with bladder cancer developed sudden dysarthria and left hemiplegia. MRI revealed occlusion of the right middle cerebral artery (MCA). Cerebral angiography revealed a large carotid free-floating thrombus (CFFT) at the origin of the right internal carotid artery (ICA) and right M1 occlusion. A balloon-guide catheter (BGC) was directly guided distal to the CFFT. Mechanical thrombectomy (MT) was performed on the M1 occlusion while the balloon was inflated to block antegrade blood flow, and good recanalization was achieved. To continue processing the CFFT, the deflated BGC was pulled to the common carotid artery, and the thrombus dispersed into the external carotid artery (ECA). Subsequently, the patient’s symptoms improved. Directly advancing a BGC distally to a CFFT may be a useful treatment strategy for tandem lesions with carotid free-floating thrombi.

## Introduction

The treatment strategy for carotid free-floating thrombus (CFFT) has yet to be established. Although the efficacy of carotid artery stenting (CAS), mechanical thrombectomy (MT), or carotid endarterectomy (CEA) has been reported in small cases [1-3], those with acute ischemic stroke with a tandem lesion due to CFFT treated with neuro intervention remain unelucidated. Therefore, we report a case of acute ischemic stroke having a tandem lesion with CFFT treated successfully by a novel technique-direct advancement of a balloon-guide catheter (BGC) distal to the CFFT. The thrombus located at the carotid artery had dispersed into the external carotid artery (ECA).

## Case presentation

**Figure 1:**
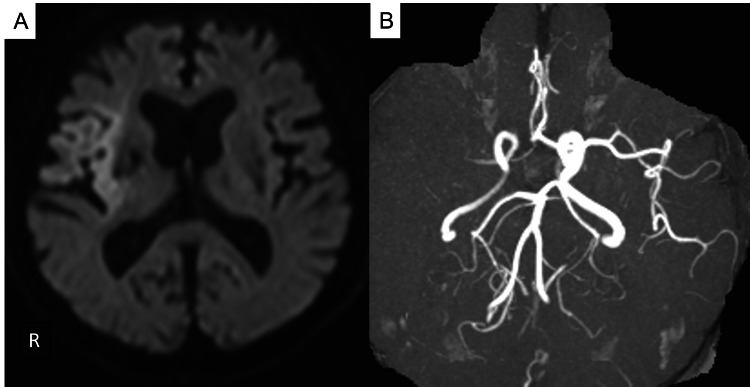
MRI on admission MRI showed a slight diffusion-weighted imaging (DWI) high intensity in the right hemisphere (A) and M1 occlusion of the right middle cerebral artery (MCA) (B).

A 68-year-old man was admitted to the hospital for chemotherapy of bladder cancer. During the admission, he suddenly developed left hemiplegia and left unilateral spatial neglect. His National Institutes of Health Stroke Scale (NIHSS) score was 17. Diffusion-weighted magnetic resonance (MR) imaging showed acute ischemic lesions in the right hemisphere (ASPECTS-DWI:7) and M1 occlusion of the right middle cerebral artery (MCA) (Figures 1A-1B).

**Figure 2:**
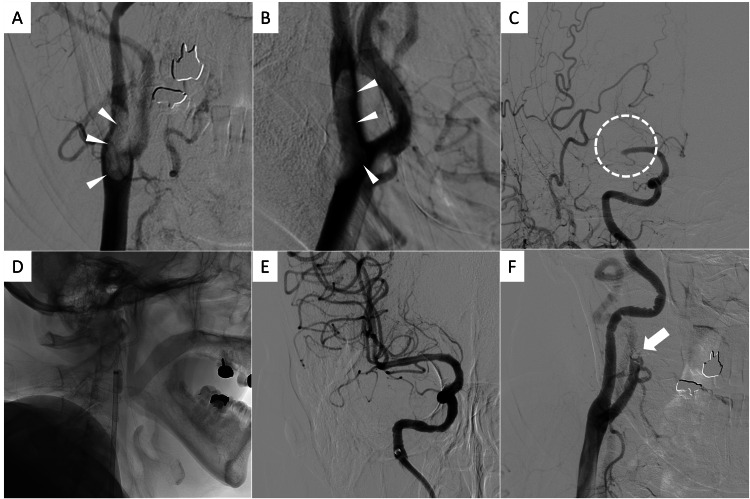
Angiography and mechanical thrombectomy The first angiography revealed a large carotid free-floating thrombus (CFFT) (A, B; arrowheads) and an M1 occlusion (C, dot circle) (tandem lesion). The balloon guiding catheter (BGC) was advanced directly distal to the CFFT to block the antegrade blood flow (D), and intracranial mechanical thrombectomy was performed. M1 was quickly recanalized (E), and the floating thrombus had migrated to the external carotid artery when the BGC was pulled to the common carotid artery to treat the CFFT (F; arrow).

Emergency cerebral angiography revealed a large CFFT located at the origin of the internal carotid artery (ICA) and right M1 occlusion (tandem lesion) (Figures 2A-2C). The right anterior cerebral artery (ACA) A1 segment was hypoplastic (Figures 2B-2C). We considered retrieving the CFFT first by aspiration or stent retriever, but there was concern that the BGC would be clogged because of the large thrombus resulting in a longer procedure time and distal migration of the thrombus during the retrieving. Moreover, recanalization of the occluded MCA should have been prioritized to save the penumbra tissue. Thus, we decided to navigate BGC directly distal to the CFFT carefully. We advanced along the CFFT side slowly and carefully with a 0.035-inch Radifocus guide wire (Terumo Co, Tokyo, Japan) with 4 to 6-Fr Countdown JB2, inner catheter (Medikit Co, Tokyo, Japan) in the 9-Fr Optimo, and BGC (Tokai Medical Products, Aichi, Japan) coaxially to minimize the catheter ledge. The balloon was inflated sufficiently distal to the thrombus to block the prograde blood flow (Figure 2D). The M1 thrombus was retrieved by EmvoTrap (Cerenovus, Johnson & Johnson Medical Devices, Irvine, California, USA) and Catalyst 6 (Stryker, Kalamazoo, Michigan, USA) with combined technique resulting in good recanalization (Figure 2E). Next, we attempted to treat the CFFT. To prevent distal embolization, SPIDER FX 5 mm; (Medtronic, CA, USA), filter protection, was deployed in the petrosal portion of the ICA and then, the BGC was deflated and pulled down to the common carotid artery. Angiography after placement of the filter showed that the floating thrombus had migrated to the ECA, and the right ICA was recanalized (Figure 2F). The procedure was finished. After the procedure, a rapid improvement of the neurological deficits was obtained: the NIHSS improved from 17 to 3 points. The patient was in a good clinical course and the modified Rankin Scale after three months was 2.

**Figure 3:**
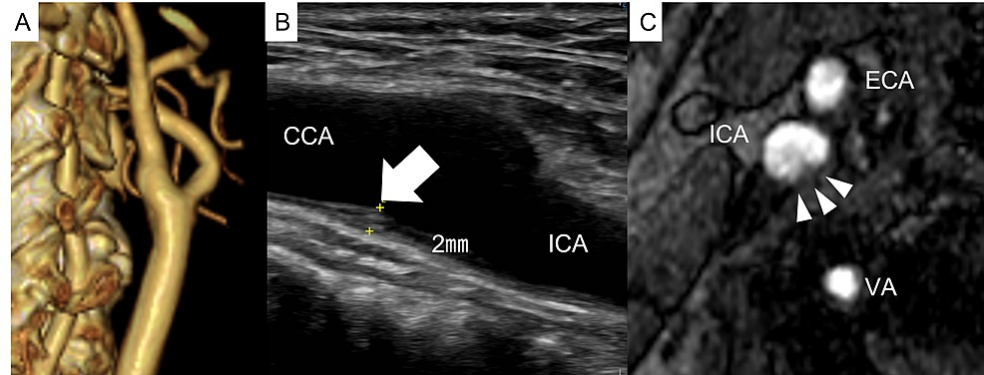
Detailed carotid artery examination after carotid free-floating thrombus dispersal A: Contrast-enhanced CT angiography showed smooth vessel wall and no stenosis; B: Carotid duplex showed a small plaque of approximately 2 mm in diameter at the bifurcation; C: Time-of-flight magnetic resonance angiography also showed a small plaque at the bifurcation. CCA: common carotid artery; ICA: internal carotid artery; ECA: external carotid artery; VA: vertebral artery.

Thorough examinations were performed to determine the cause of CFFT. Aggressive blood test, including coagulopathy, was negative except that the D-dimer and CA19-9 were increased, 4.1 μg/ml (<0.5 μg/ml) and 217.4 U/ml (<37 U/ml), respectively. The carotid artery lesions showed almost smooth vessel walls on final angiography, as seen in Figure 2F, and contrast-enhanced CT after the procedure showed that after the floating thrombus had dispersed (Figure 3A). However, carotid duplex ultrasonography and time-of-flight (TOF) MR angiography showed a small plaque lesion at the bifurcation (Figures 3B-3C). Holter electrocardiogram showed failed to detect any atrial fibrillation. Contrast-enhanced CT showed a venous thrombus in the lower extremities, but transesophageal echocardiography did not show a right-to-left shunt. Finally, we diagnosed that the CFFT was caused by a coagulation abnormality associated with bladder cancer, which led to the formation and growth of a local thrombus anchored by a small plaque in the carotid artery. Apixaban 5 mg b.i.d. was introduced as an antithrombotic drug, and the patient passed without recurrence.

## Discussion

CFFT is rare, as fewer than 150-200 cases have been reported to date, and it occurs in < 1% of cases of cerebral ischemia [1,3]. The clinical nature and management of stroke associated with CFFT have yet to be established yet. There are previous reports of CFFT treatment with CEA [1] or endovascular treatment (stenting or thrombectomy) [2,3]; however, there have been very few reports on hyperacute tandem lesions in association with CFFT. To our knowledge, our study is the first to report tandem lesions with CFFT, treated by our novel method: direct advancement of a BGC distal to the CFFT. The thrombus located in the ICA disappeared and was dispersed into the ECA when the BCG was pulled down to CCA.

In cases of tandem lesions, it is controversial whether to treat the intracranial or proximal lesion first [4,5]. When considering the treatment strategy for this case of a tandem lesion with a large CFFT, we considered treating the proximal CFFT first and then treating the intracranial lesion. However, there was a concern that the BGC would be clogged by the large CFFT, and that it would be torn off and scattered to the distal by stimulating the CFFT, then, the intracranial cerebral infarction would be enlarged due to the time required for the treatment of the proximal lesion. On the other hand, direct distal advancement of the BGC posed the risk of spreading floating thrombus and plaque. In this case, the ipsilateral A1 was hypoplastic and the M1 was already occluded, so much even if the CFFT migrated to the distal side, there was little chance of embolization to new territory, and we judged that the thrombus could be retrieved together. The carotid artery itself was not stenotic, and to perform intracranial treatment in a shorter time, we decided to guide the BGC directly to the distal to CFFT. After MT of the occluded M1, the BGC was pulled down to the common carotid artery, and angiography revealed that the floating thrombus had migrated to the ECA. Contributing factors could have been that the BGC blocked the antegrade blood flow into the ICA, which might have guided the thrombus into the ECA, or that the inflated BGC was moved by the recoil of the aspiration catheter, which acted like a Fogarty catheter [6]. We believe the strategy of advancing the BGC distal to the CFFT to shorten the procedure time of the intracranial treatment is an effective strategy for the treatment of tandem lesions with CFFT.　

Although most CFFTs are caused by atherosclerotic lesions, there are also reports of CFFTs caused by dissection, trauma, vasculitis, and coagulation abnormalities due to malignancy, pregnancy, infection, and inflammation [7]. In this case, thorough examinations found only a small plaque at the bifurcation of the ICA. CA19-9, which was elevated in this case, is elevated in pancreatic cancer and ureteral epithelial carcinoma, such as bladder cancer [8].CA19-9 exists in the blood as a sialomucin macromolecule, and its sialic acid residues directly activate prothrombin in the blood [9], suggesting that CA19-9 itself contributes to thrombosis. We hypothesized that the micro-plaque lesion in the carotid artery could be a foothold for the local formation of thrombus due to hypercoagulation caused by the tumor. Cancer-related cerebral infarction (so-called Trousseau’s syndrome) is often characterized by multiple small infarcts in multiple vascular territories [10], but our case showed that strokes due to CFFT could develop as cancer-related cerebral infarction.

## Conclusions

Tandem lesions with CFFT were successfully treated by advancing the BGC directly distal to the CFFT. The CFFT consequently moved to the ECA. This strategy may be effective for hyperacute tandem lesions with CFFT. We also showed that CFFT-induced cerebral infarction may develop as cancer-related cerebral infarction.
